# Harm reduction and “Clean” community: can Viet Nam have both?

**DOI:** 10.1186/1477-7517-9-25

**Published:** 2012-07-09

**Authors:** Thu Hong Khuat, Van Anh Thi Nguyen, Melissa Jardine, Timothy Moore, Thu Huong Bui, Nick Crofts

**Affiliations:** 1 Institute for Social Development Studies, Suite 225, Entry 11, CT5 Building, My Dinh-Song Da Area, Pham Hung Road, Ha Noi, Viet Nam; 2 Nossal Institute for Global Health, University of Melbourne, Melbourne, Australia; 3 School of Population Health, University of Melbourne, Melbourne, Australia; 4 Centre for Law Enforcement and Public Health, Melbourne, Australia

## Abstract

The findings of our research show that while police play multiple roles in the fight against drug-related crime, they often perceived their tasks – especially preventing and controlling drug use on the one hand, and supporting harm reduction on the other – as contradictory, and this creates tensions in their work and relations with their communities. Although they are leaders and implementers of harm reduction, not all police know about it, and some remain skeptical or perceive it as contradictory to their main task of fighting drugs. Methadone treatment is seen by some as in competition with their main task of coordinating conventional drug treatment in the rehabilitation center.

The history of drug use and the evolution of discourses on drug use in Viet Nam have created these conflicting pressures on police, and thus created contradictory expectations and led to different views and attitudes of police regarding various harm reduction measures. This might aid understanding why, despite the comprehensive and progressive policies on HIV/AIDS and harm reduction in Viet Nam, it is not easy for police to actively and effectively support and be involved in harm reduction at the ground level.

To promote the wider acceptance of harm reduction the concept of *community safety* must be expanded to include *community health;* harm reduction must be integrated into the “new society” movement; and laws and policies need further revision to reduce contradiction between current drug laws and HIV laws.

Harm reduction guidelines for police and other actors need to be disseminated and supported, embodying better ways of working between sectors, and all sectors in the partnership require support for building capacity to contribute to the overall goal.

## Introduction

Viet Nam has had a major concentrated epidemic of HIV, from detection of the first case in 1990 to an estimated 280,000 infected in 2012, approximately 0.47% of the population. Around 65% of those infected have histories of injecting drug use (IDU), and among them HIV prevalence has reached 70% or more [1]. As detailed elsewhere in this volume, Viet Nam has responded with development of comprehensive and progressive policies on HIV and AIDS, including policy and legal support for harm reduction [2].

How effectively do these policies translate to street level; and do they actually enable the work of implementers of harm reduction approaches? And more specifically, given that the major mechanism of implementation of laws is through law enforcement, especially by police: how effectively is law enforcement, especially community-level policing, involved in harm reduction in Viet Nam, and how can this involvement be improved?

We here examine the involvement of law enforcement in harm reduction in Viet Nam, illustrating the experience of police at street level, to make recommendations about how the police role can be more effective. This research was part of a broader program of research entitled “*The Impact of Harm Reduction Programs on Law Enforcement in Southeast Asia: What Works and What Doesn’t”* administered by the Nossal Institute for Global Health in partnership with the Institute for Social Development Studies (Vietnam), the National Institute of Public Health of Cambodia and Laos University of Health Sciences.

## Background

### Current situation of drug use and HIV in Viet Nam

As of 30th November 2011, there were 158,414 drug users recorded nationwide, an increase of 5.7 percent compared to the same time in 2010; this figure reflects only those registered – it is unknown how it relates to the actual number. Heroin was the most popular drug used in Viet Nam, predominantly injected [3]. About 40,000 drug users, 18.2 percent of those registered, are at any one time in 121 rehabilitation centers across the country [4].

Since the first case of HIV was detected in Ho Chi Minh City in 1990, Vietnam experienced a growing epidemic of HIV. Nationwide, by the end of 2011, there were 197,335 people living with HIV, of whom 48,720 had developed AIDS and 52,325 had died as a result of AIDS. The major factors driving the epidemic include the sharing of injecting equipment among injecting drug users (IDUs), with IDUs accounting for 43.1% of the total number of infected individuals. HIV prevalence among IDUs in 2011 was 13.4%, down 3.8% compared to 2010, yet still the highest of any group since the early 1990s. In several provinces, the proportion of IDUs among HIV-infected people is as high as 65 to 70%. Men who have sex with men (MSM) have the second highest national prevalence rate, at 5%, followed by female sex workers (SWs) with a rate of 3% [5]. There is overlap between IDUs and sex workers, with high proportions of IDU among sex workers in some cities of Viet Nam [5].

However, while still considered a ‘concentrated’ epidemic primarily affecting IDUs, female SWs and MSM, in some areas HIV is spreading to the general population through chains of sexual contact involving male IDUs and their female sexual partners, and sex workers and their clients, associated with an increasing overlap of the two groups. While male cases still predominate, the proportion of HIV-infected people who are female is increasing each year. In 2011, females made up 31% of all those infected, up two percent from 2010.

### Harm reduction

By the end of 2011, harm reduction programs involving provision of sterile needles and syringes have been implemented in 60 provinces and cities, while condom distribution occurs in all 63 provinces and cities. More than 30 million needles and syringes were distributed by peer educators to drug users and about 28 million condoms were distributed free of charge to populations with high risk behaviours in 2011, an increase of 11.2 percent and 12 percent respectively compared with 2010.

By the end of 2011, methadone maintenance therapy (MMT) has been implemented in 11 provinces and cities in 41 centers for 6,931 patients with positive results in various aspects including improvement in health and well-being, improved measures of cost effectiveness, and reduced crime and family conflict [3]. The government has decided to expand the MMT program to 30 provinces and cities by 2015. Anticipating the decrease of international support in the coming years as Viet Nam has reached the classification of Middle Level Income country, the Ministry of Health is now drafting plans to produce methadone locally to meet the demand of hundreds of thousands of drug users. This plan is to be submitted to the Prime Minister for approval during 2012.

Taking into account the fact that the first pilot harm reduction activities were in 1993, nineteen years ago, and the multiple but unsuccessful efforts to advocate for substitution therapy, specifically MMT, for years, one may raise the question of why harm reduction has only recently been expanded widely … and why it took Viet Nam such a long time to accept it?

## Methods

After an intensive review of all policy documents and research literature on illicit drugs, HIV infection and AIDS, and harm reduction accessibility in Vietnam, a consultation workshop was held with experts and representatives of agencies working on harm reduction in Viet Nam, who helped to provide the research team with an overview of the situation of drugs, HIV infection and AIDS, and harm reduction in Viet Nam and suggested a list of potential research participants. With this background, we conducted interviews in Ha Noi between April and October of 2011; the research participants were policy makers, program managers and police working on drugs, harm reduction and HIV infection and AIDS at different levels from national and city down to community level, and community leaders. A total of 58 people participated in the interviews and focus group discussions: of 40 people interviewed individually, 20 were police at district and commune level; as well, 27 local authorities and leaders of social organizations from three communities of Ha Noi took part in three focus group discussions.

The participants were categorized as policy makers, program managers, police and local leaders. Interview question guides and discussion guidelines were then developed for each category of the participants. The research instrument and protocol were reviewed and approved by the Internal Review Committee of the Institute for Social Development Studies. Each participant was contacted in advance by telephone and informed about the purpose and the procedures of the research. Before interview, verbal consent was obtained after participants were informed about confidentiality of their personal identification and that their participation was voluntary and could be ceased at any time. Each participant was given a gift valued about VND100,000 (USD5) as acknowledgement for his or her time. On average, each interview lasted about an hour and each focus group discussion took two hours or more. Most interviews and focus group discussion were taped, with permission of the participants.

Interviews and focus group discussions were then transcribed and categorized. The data analysis included developing codes, identifying themes and connecting the themes.

To ensure confidentiality, each research participant has been given a fictional name, and information about their work place has been kept broad.

## Results

### Police at street level –“between the devil and the deep blue sea”

Here we examine the involvement of police in harm reduction in Viet Nam, illustrating their position through the experiences of the police officers.

Through their stories, we found police at street level are caught in a difficult position. On the one hand, they have to fulfil the tasks assigned by their superiors; on the other, trying to fulfil those tasks, they risk going beyond or against other policies … or if they follow these other policies, they risk losing trust of the community, who expect them to maintain security and social order in the neighbourhood.

#### Fulfiling the police role in drug treatment - “meeting quotas is very stressful”

Fighting against drugs is seen as crucial for the socio-economic development of the country. In this battle against drugs, police are assigned a key role which is reflected in various legal and policy documents. For instance, the Drug Law 2000, article 38, stipulates that police have “to direct the detection of drug users and the arrangement of sending them to the compulsory drug treatment establishments, keep security and order in these establishments, control drug treatment in community and in drug treatment establishments” [6].

The Decision 61/2000 TTg by the Prime Minister on the establishment of the National Committee on AIDS, Drugs and Prostitution defined the role of Police as a standing agency in preventing and combating drugs; with responsibilities

· to coordinate, organize and lead implementation of multi-sectoral program on prevention, combating and controlling drugs nationwide; and

· to gather, categorize drug users and sex workers to send them to treatment establishments in accordance with the Ordinance of Administrative Violations.

At the grassroots level, in those communes affected by drug problems, the commune president can set up a communal board to implement activities for prevention of AIDS, drugs and prostitution in the community [7]. One Vice-president of the Commune is the Chair of this Board, and the Vice-head of the Commune Police is appointed as Vice-chair, in charge of the drugs issue. For this reason, when we contacted local authorities in Ha Noi for this research, we were often referred to meet with the Vice-head of the Commune Police. From interviews with those police officers we learned a great deal about how police at street level are involved in drugs issues, including drug treatment and harm reduction.

The first police officer we met is An, a Vice-head of a police station of a ward^a^ in the northern part of Ha Noi, one of the drug hubs of the national capital. He is also a Vice-chair of the Board for prevention of AIDS, drugs and prostitution of his ward. An described the work of police at the street level:

“Regarding the work of the ward police, there are two major tasks. First, fighting against drug related crimes such as drug smuggling and trafficking; second, controlling drug users in the ward and sending them to rehabilitation centers for treatment. Police always play a key role in the fight against drugs.”

An was very proud of the task of ensuring security and social order in his ward. Detecting and fighting against drug crimes and controlling drug users is an important part of maintaining social security and order in the community and thus contributing to the protection of the society:

“The police task is to protect the Party, the Government and people. I am responsible for all security matters in this commune including prevention and combating drugs and supervising drug users. I report to the communal party unit and local authorities and recommend the measures to deal with drug users within our community.”

An said his community used to be a hot spot in terms of drug problems in the city a few years ago, with more than 100 drug users and dozens of drug smuggling spots. However, the situation has been controlled and gradually improved. He believes that to maintain this achievement, three activities must be well implemented simultaneously: education, combating drug trafficking and drug treatment and post-treatment rehabilitation, including employment support. Police were assigned to coordinate the implementation of both drug treatment measures: community-based voluntary treatment, and compulsory detention in the rehabilitation center. However, An believed that community-based treatment was ineffective and resource wasteful.

Regarding the task of police in coordinating drug treatment, An described it thus:

“Police control [the] situation [in the ward], particularly the number of all drug addicts, and every month to have meeting with them to educate them and warn them that they should not relapse. If they show signs of relapsing they would be educated within the community for about six months. If they fail, then the ward police compile their record for sending them away [to a rehabilitation center].”

The routine of the police at ground level includes not only fighting drug crime and controlling drug use in their community, but also supervising those who have returned from an 06 center^b^. Nam, a police officer in a residential cluster of a district in the Southern part of Hanoi said:

“Regarding those who return from the center, we invite them to the ward police station, ask them to write a commitment not to use drugs again. Every quarter, they have to come here to take a test. If they relapse, they need to be educated. Those who keep using drugs will be arrested. That is the duty of ward police.”

According to Tan, a Vice-head of police from another district which is an important hub of the drug trade in the center of Ha Noi, his district has a larger number of drug users as compared to other districts of the city. He said that in this district there were almost 2,400 drug users, about 1,000 to 1,200 of them reintegrating in the community, having left the 06 centre. Tan said:

“To prevent HIV infection and reduce crimes there is no other way better than controlling drug users. There are various controlling measures. First, to send to 06 center those drug users who met criteria of compulsory treatment. Second, to help sending those who can afford to pay voluntary treatment to the center. Lastly, to organize and supervise community-based treatment.”

However, the tasks of police at street level regarding drug treatment are not simple, and are often stressful because they have to meet quotas of drug users to be sent to rehabilitation centers. According to an official from the Center for AIDS Prevention of Ha Noi, each year the Department of Labor, Invalid and Social Affairs (DOLISA) of Ha Noi City assigns every district a quota of 200 to 300 drug users to be sent to rehabilitation centers. The District Office of Labor, Invalid and Social Affairs divides the quota to the wards or communes, depending on the perceived situation of drugs in the wards.

Talking about the quota, Huy, a police officer from a southeast area of the city, said:

“This year [the] District Office of Labor has assigned a quota of 210 drug users to be sent to rehabilitation center. The District Office distributes the quota to wards. My ward, for instance, gets a quota of 20 drug users. You do not fulfil your task if you do not meet the quota, which means you would be ranked B or C only. It is very stressful to meet the quota.”

Huy complained that the quota is too high for his ward, and explained that it is hard to meet the quota, not because there are few drug users in his ward, but because of constraints which come from the Decree 135 [8]. According to this Decree, drug users who have relapsed after time in the rehabilitation center can only be sent to the center again after 24 months from the day they were released from the center. Within this period of time, the drug user is to undertake drug treatment in the community for six months, in accordance with Resolution 163 on measures of education in the community for drug users [9]. At the time of interview in his ward there were 94 drug users but they did not fit the criteria of Decree 135, as all of them had left the center less than 24 months previously. Huy was therefore unable to meet the quota of 20 drug users to be sent to the center, but had to process a lot of paper work to arrange community-based treatment for those 94 drug users. Huy wished to recommend revision of Decree 135 so that those drug users who tested positive would be sent to the center immediately, regardless of how long they had been out of the center. He believed that education and treatment in the community was wasteful, because all drug users relapsed, some even returning to drug use immediately after release from the center. Huy felt very anxious because when there were too many drug users in the community they could commit bad things and he would be blamed for not fulfiling his task, especially if the drug users of his ward were caught in the neighboring ward.

Phan, a police officer in a ward in the old quarter of Ha Noi, said his ward has been assigned a quota of 27 to 30 drug users to send to the rehabilitation center each year. This number could be distributed to all residential clusters in the ward depending on the prevalence of drug users in each cluster. In 2010 he was able to send three drug users to the rehabilitation center. However, he said

“Honestly speaking, we have to meet the quota but not because of that we could send any drug user to the center. We have to re-educate them, persuade them for a while and can only arrest them when those measures failed. It is not necessary to meet the quota of 30 if there are people who do not fit the criteria.”

Long, a police officer in the southeast area of Ha Noi said it was often very stressful for him to meet the quota. In 2008, he was assigned to send 49 drug users to the center but he could send 23 people only. He felt bad because he could not fulfil his task. However, Long raised another difficulty in meeting the quota. It was not easy to send drug users to the center because their families often did not co-operate with the police. The drug users could run away before they were caught. Long said:

*“Their family is miserable with the drug user but often does not support the police if we come to take him to the center. So that when we receive a letter*^c^, *we have to come to his house immediately and take him right away. He would run away if we inform him in advance. One police officer reads the letter, the other has to handcuff him right away. This year, if we are assigned a quota of 5 we have to find 5 by all means.”*

This also happened to An. Some weeks previously, a man came to see An and begged him to take his son away. An and his colleague spent two days tracing the young man and finally caught him when he was injecting drugs. However, when the documents were being processed, his father came back and again begged An to release his son. The father wanted to help his only son to undertake treatment at home.

Not only being stressed by the need to meet a quota, the police reported also feeling low-spirited sometimes. Almost all the police we interviewed shared the same feeling as Nam:

“… Our ward is strong during the last few years but only strong in sending [drug users] to the center. In fact, very few of them can quit drugs … Therefore controlling and supervising drug users is very tiring. This is a thorny problem for us. I work with this problem for many years but it remains unsolved.”

Phan feels very sorry for drug users and their families, but he believes that sending drug users to rehabilitation centers is better for both drug users and their family:

“In general, it is miserable. You would feel sorry for them if you know their life. Both drug users and their family suffer. It is very sorry to see a drug user who from morning until noon keeps eating a bowl of rice but cannot finish it. Some drug user was chained by their family like a dog. It is very pitiful but if he was unchained, he would commit bad things. I agree that drug users cannot be seen as criminals because their family often ends up in a miserable situation. Sending drug users to the rehabilitation center for two years may keep them alive but they would die if they stayed home.”

The police are often caught trapped between the regulations of policy and the expectations of their community. Anxiety and complaints from people in the community about drug users are an additional pressure on police. Huy felt bad because he failed to keep security in his ward. Because of regulation of the Decree 135, he could not send even one of the 94 drug users in his ward, although many of them had relapsed. Huy said:

“Among those who returned from the center I know at least 60 to 70 percent of them relapsed. Some of them even relapsed right after being released from the center. What can we do with those guys? When they tested positive, I passed them to post-treatment program run by the Women’s Union and the Youth Union or the War Veteran’s Association who are keen to help them to be good people again but they failed to be good people. Their parents were also hopeless about them. The police tried hard to educate them but they did not change. People in community anxiously ask “Why don't you send them to the center?”

Regardless of how hard their work is in sending drug users to the rehabilitation centers, like Hung, cited below, many police officers believed that it is better to send all drug users to the centers and to keep them there for a long time, because:

“Those guys are, anyway, already addicted. We just want for our community to be clean and peaceful. If we let these guys stay home, a lot of problems happen daily, like petty theft or stealing money or things. It is very stressful. Sometime, I was assigned a quota of 1 or 2 only but those guys who are really bad would also be taken.”

According to Hien, a colleague of Hung, compulsory treatment is the optimal approach because he believed it provided treatment to drug users and keeps them away from drugs. So Hien believed that all drug users including those who relapsed soon after they left the center should be kept there. Many drug users died at home because of overdose, so being in the center can also save their lives. Hien said that to reduce the stress and burden for police it is better to send those drug users who tested positive to the centers immediately, to prevent them from running away. The police sometimes have to trace runaway drug users as far as Hoa Binh province. He found this insecure and costly. At the time of the interview Hien said he still owed one person from his quota because a man ran away after a positive urine test.

An on the other hand thought it impossible for IDUs to get off drugs. On average, out of 100 drug users, after detoxification 98 relapsed, one was put back into jail and one was found dead. He said:

“I see that needles and syringes are very cheap and easy to buy now in Vietnam, if not free for drug users. As far as I know, they often buy new syringes every time they want to inject, together with a pack of drug and some water. However, because more often than not they don’t have enough money they tend to share one dose among three people using just one syringe and needle. They may know they should use clean needles and syringes but because of their tight economic situation, and the relatively high cost of drugs, they rarely do so.”

Being Vice-head of the Community Standing Board for the prevention of AIDS, drugs and prostitution, An should quite properly be concerned about this, as the HIV epidemic in Vietnam HIV is driven by drug injection.

In summary, controlling drug users and sending them to rehabilitation centers are major tasks of police at street level. However, these tasks can be stressful for them because of conflicts between the quota system and policies such as Decree 135 and Circular 163, community attitudes and pressures and the lack of collaboration or negative attitudes of some families of drug users.

#### Doing harm reduction: “showing the path for the deer to run away?^d^”

Harm reduction was introduced to Viet Nam in 1993; however it remained as pilot activities in limited areas of the country for more than fifteen years, before harm reduction was officially accepted by the National Strategy on HIV/AIDS Prevention and Control in 2010, in its vision to 2020. Nevertheless, more intensive harm reduction programs could only be expanded after the issuance of the HIV/AIDS Law 2006, which permits the implementation of harm reduction and stipulates general principles for harm reduction. One year later, in 2007, Decree 108 provided further guidance for harm reduction activities to be implemented nationwide. Soon after, the National Action Plan on harm reduction for HIV prevention in 2007–2010 set targets and indicators for implementation in all provinces and cities of the country. The Revised Drug Law 2008 regards drug users as victims or patients and thus deserving of treatment, further reinforcing harm reduction.

The role of police as leader and implementer of harm reduction intervention programs in collaboration with the Ministry of Health and relevant agencies was defined by the National Action Plan on Harm Reduction.

But in practice, not all police know about harm reduction. Lu, a policeman from the ward in the southwest area of Ha Noi, said he has never heard the term “harm reduction” and has not yet been assigned any task related to the distribution of needles and syringes or condoms:

“I have never participated in harm reduction activities. Police in our ward never have been mobilized to take part in any activity like condom distribution. Probably this is assigned for mass organizations. We are not involved in this. Our regular task is to just educate and supervise drug users.”

Lu, however, was skeptical about programs of supplying needles and syringes after it was explained to him what this meant. He advocated increasing the time drug users stay in the rehabilitation centers and believed this would be better than carrying out harm reduction activities.

When asked what he would do if he saw a woman giving a clean syringe with needle to a drug user, Phan said:

“You say she distributes the syringe and needle for him to inject drug? No, it is impossible. This means to encourage him to use drug. It can be tolerated in case if he is in the latest stage of AIDS. However, for a drug user who is trying to quit drugs, giving him a syringe and needle may make him come back to drugs. This means showing the path for the deer to run away. This means the government allows him to use drug, then how the police can arrest him? No, I don’t accept that.”

Like Phan, Ba, a policeman from a residential cluster of the central district of Ha Noi, and other policemen interviewed also believed that providing needles and syringes is encouraging drug use:

“Providing syringe and needle to IDUs is said to prevent HIV. However, in fact, this looks like encouragement of drug use. Meanwhile police have to force them to go to the compulsory drug treatment center … In my personal opinion the provision of needle and syringe is a sort of encouragement.”

Lam, a policeman from the southern area of Ha Noi, said supplying needles and syringes to drug users is unreasonable. He would not want to take part in this activity because he did not want to be misunderstood by the community:

“If I provide syringe and needle to drug users, their families would protest, saying you police facilitate drug use. Therefore I’d rather not be involved in this activity.”

Long understands that distributing clean needles and syringes is for HIV prevention, but he sees this as encouraging the use of drugs. He found this contradictory to his task of sending drug users to rehabilitation centers.

Chien, a high ranking police officer at the Ministry level, while strongly supporting harm reduction, is also fully aware of the concern of people in the community about the needle and syringe program:

“In several provinces, war veterans and retired officers as well as community people did not support this program. In a northern province, a strong campaign was launched to mobilize people to detect drug users and to send them to rehabilitation center, then they found this syringe and needle program contradictory to their efforts in keeping the community clean. In the meetings of war veterans or retired officers, many of them expressed disapproval.”

Tan, the Deputy Head of the district police considered the police role as: being supportive but staying behind:

“… regarding the provision of syringe and needle, the ward health center is instructed to collaborate with peer educators – who used to be drug users. Police are better to stay aside, because the drug users are afraid [of police]. Therefore, although we support this, we stay behind.”

Tan has openly appreciated methadone therapy and said he would support this program in his district:

“If IDUs change to use methadone, I think this should be effective. We would gather IDUs to inform them about the effects of methadone: it is cheap; like a normal medicine, if taking it every day one would no longer crave for drugs, especially it has no risk of HIV infection, meaning safe addiction. Being told that, they would be happy to use methadone. I think 100 percent of them will like to use methadone… We were waiting for this program for years but it never comes.”

However, he suggested that police must be closely involved in the process with strong measures of a punitive nature:

“I already said to the steering committee that implementing methadone program will be impossible without police’s hands. At first, police must be involved, then it will become routine … Because from the beginning, IDUs may not be comfortable, they would be scared … Police would bring them in, one or two times, ask them to write a commitment. Those who violate the commitment will be immediately sent to [rehab] center by the police, as an example for others.”

Phan confirmed that he would strongly disagree with the supply of needles and syringes for drug users but he accepted drug treatment with substitution of methadone. He believed that once methadone became available, the police burden would be relieved:

“I agree with methadone. We will be more relaxed, won’t we? I hope methadone will be accessible in my ward. It is not as harmful as heroin, is it? I support methadone. It is good and should be made available to drug users.”

Hung was, on the other hand, skeptical about the effectiveness of methadone. He did not think that methadone could help those who were heavily addicted to heroin to quit. Hung thought that if the harsh measures of conventional drug treatment did not help then methadone would also fail.

Son, Vice-Head of police in a ward in a northwest area of Ha Noi, expressed his concerns about security in the community if drug users, instead of being sent away to the rehabilitation centers, were to stay in the community for methadone treatment:

“My greatest concern is for security and order in our community. When drug users change from heroin to methadone and are no longer to be sent to the center, what if there are thefts or disorder? Implementing the methadone program means the number of drug users sent to the rehabilitation center will be reduced, but how can I certain that those who use methadone will be able to quit heroin? Because the methadone is too light for those who are heavily addicted to drugs, who knows what would happen during this process? I think methadone is not suitable for those who are heavily addicted.”

The officer of the Center for AIDS prevention revealed that in some districts, police still were concerned about the quota for the centers:

“In one district, in the meeting to prepare for implementation of methadone treatment, some police said they have to meet the quota so they would send drug users to rehabilitation center but not to methadone treatment.”

Long openly confirmed that he would rather prioritize meeting the quota than “sharing” drug users to the methadone program because he wanted to fulfil his major task and because he was not yet convinced about methadone.

Han, Vice-head of police in a ward of a northwest district of Ha Noi, where a pilot methadone program has been running since 2010, was highly appreciative of this new method of drug treatment. He was convinced about the benefits of the program but wondered about its sustainability. He was concerned how long the program would last, and if the program ceased because the budget was no longer available, if the participants would return to drug use again. Han was also concerned about the “competition” between the methadone program and the conventional compulsory drug treatment in the center and sometimes, because of pressures of the quota, how police would prefer to send drug users to the rehabilitation centers rather than encouraging them to join the methadone treatment.

A high-ranking police officer at the national level stressed the importance of consensus among community members on various measures of harm reduction. He said police do not want to do things of which the community is still not supportive. He was also concerned about sustainability of the methadone program. He said:

“We police are very closely attached to community. We spend 24 hours a day with people, taking care of security matters, convincing community members to support the methadone program. Let’s say, when people are happy about the program it is terminated because resources are no longer available. Then we police become liars. We lose people’s trust. It is the worst of worst things because we need people’s support.”

The officer of the Ha Noi AIDS Center expressed a similar concern:

“We all know that compulsory drug treatment in 06 centers and methadone treatment are simultaneously implemented. Those people who are heavily addicted and frequently relapse often create troubles in the community. They are of course the most wanted for the compulsory treatment in the 06 center. This obviously affects the methadone program. Many drug users want to join methadone treatment but they do not dare to disclose themselves because they are afraid of being sent to the 06 center - so they do not register for the methadone program. This is a challenge to our program. It is difficult to have clarity in this situation, because both programs target drug users. There is a dilemma that the heavily addicted people are the most wanted for compulsory treatment in the center while these people are also of the priority of the methadone program.”

In summary, although they were assigned the role of leader and implementer of harm reduction programs, not all police we interviewed knew about it, and some were still skeptical about the effects of the program or perceived it as contradictory to their main task of fighting drugs. They saw supplying needles and syringes as encouragement of drug injection – “showing the path for the deer to run away”. Some police even see that support for methadone treatment would compete with their role in coordinating conventional drug treatment in the rehabilitation center.

## Discussion

It is clear from the stories of An and the other police that the difficulties they face in their working lives come about because of their conflicting tasks - detecting, arresting and putting drug users into 06 centers while promoting harm reduction. An and his colleagues feel trapped being fighters against drug users on the one hand, and supporters on the other. Why are the police caught in such a difficult situation?

In an attempt to answer this question we reviewed the history of drugs and the way drug use has been constructed and addressed in Viet Nam (presented elsewhere in this volume). We found that the discourses of drugs have been evolving over time, though there was no clear line between various discourses, but rather some overlapping. We also realized that understanding drug issues is impossible in isolation from the broader socio-political context of the country.

Working in such a web of overlapping and sometimes conflicting policies, police at street level find themselves inextricably entangled and conflicted, as is reflected in their narratives. On one hand, being in charge of keeping security and maintaining social order in the community, they devote themselves to their tasks of fighting against drugs. On the other hand, being aware of the threat of HIV transmission, many do appreciate the importance of harm reduction. They are torn between the pressure of meeting their quotas and the expectations of their community, and their wish to contribute to the prevention of the HIV epidemic. Being put in such a dilemma, it is understandable if the police prioritize fighting drugs.

In fact, underlying the conflict between policies about drugs, HIV/AIDS and harm reduction, is an ideological issue. As a war veteran we interviewed openly expressed during his interview, people do not accept harm reduction *not* because they think it is ineffective, but because of ideology. However, ideology has evolved as the discourses of drug use have evolved over time. When drug use is constructed as a legacy of colonialism or as the bad remnants of capitalism, the fight against this problem must be seen as part of the class struggle. When it becomes a side effect of the market economy, drug use turns instead to be a threat to a healthy socialist society. Lastly, when it is realized as a global issue, drug use becomes accepted as a health issue, and drug users become patients.

In this concluding section, we would like to cite an officer from National Committee of AIDS, Drug and Prostitution who confirmed to us that methadone treatment should be under the management of the health sector, and not police, because drug addiction is a health issue, and from the human rights point of view, drug users should not be viewed as ‘social evils’. She strongly believed that without harm reduction being promoted and implemented in Viet Nam the HIV epidemic would be much more severe and the problem of drug use would be much more serious. It is also worth adding the opinion of the high-ranking police officer that since there is no ultimate solution to drug addiction, harm reduction is the best available approach for a solution, and involvement in harm reduction activities is of benefit to police in many ways.

To promote the wider acceptance of harm reduction among police as well as among the community, first, and conceptually underpinning every other initiative, the concept of *community safety* must be expanded to include *community health.* In the Vietnamese context, harm reduction needs to become an integral component of the “new society” movement. To bring reality to this concept, laws and policies need further review and revision to reduce contradictions, especially those between current drug laws and HIV laws.

Practically, and of use to An in his multiple roles, harm reduction guidelines for police and other actors need to be disseminated and supported by An’s superiors and the community. Better ways of working between sectors on overlapping issues such as drugs, HIV, criminality and rehabilitation are needed – especially by making referral services available through enhancement of partnerships among sectors. And all sectors in the partnership – all sectors in the community – require support for building their capacities to contribute to the overall goal.

There should be further conversations with police at the grassroots level, to better understand the position in which they find themselves, and to better figure out how we can help them resolve these difficulties and be able to act effectively in concert with effective community health approaches. An remains trapped between the two approaches which are, at first, seemingly in contradiction with each other: that of public security, and that of public health. However, these two approaches should not be contradictory – there is no reason they should not be complementary; and, second, the need to strengthen the partnership between sectors, set up community consultative mechanisms, and make referral services available and effective, so as to help An and his colleagues to get out of the trap – as, because of the quota system, the *only* choice he has now is to send IDUs to the 06 camps.

## Endnotes

^a^ Ward (*phuong*) is an administrative unit at a grassroots level in the urban area. In the sub-urban or rural area it is commune (*xa*).

^b^ 06 center is ‘Rehabilitation Center’ for drug users which was established after the Resolution 06 of the Government of January 1993. These centers are under the functioning of the MOLISA. According to MOLISA 2012, currently there are 121 centers of this type operating nationwide.

^c^ The letter is from the president of the ward informing of the decision to send a drug user to the rehabilitation center.

^d^ A Vietnamese proverb, meaning to encourage inappropriate behavior by providing an opportunity/means. Literally: a [stupid] hunter creates a path for his hunted animal to escape.

## Competing interests

The authors declare that they have no competing interests.

## Authors' contribution

THK was research field director in Viet Nam and primarily responsible for writing this paper. VATN conducted the field research and contributed to analysis and writing. THB was member of the country research team and contributed to first phase of the field research. MJ contributed to field research, analysis and writing. TM was co-Investigator on the study, responsible for liaison, co-ordination and design of the research project. NC was Chief Investigator, responsible for design and supervision of the research project. All authors read and approved the final manuscript.
